# Orthopedic Challenges in Avascular Necrosis Management: A Case Report of Surgical Complications and Multidisciplinary Care

**DOI:** 10.7759/cureus.57629

**Published:** 2024-04-04

**Authors:** Ali Z Ansari, Dhruv U Patel, Shivum Desai, Adarsh Manawa, Srihita Patibandla, Kurt Kratz

**Affiliations:** 1 Orthopedic Surgery, William Carey University College of Osteopathic Medicine, Hattiesburg, USA; 2 Emergency Medicine, University of Mississippi Medical Center, Jackson, USA; 3 Pathology, Merit Health Wesley, Hattiesburg, USA

**Keywords:** post-operative pain management, surgical intervention, joint contracture, hip surgery, orthopedic implant-related infection, orthopedic intervention, prolonged hospitalization, postoperative management, hip avascular necrosis, total hip arthroplasty: tha

## Abstract

A 52-year-old woman, with a multifaceted medical background encompassing spinal cord injury, pneumonia, and recurrent hospitalizations, presents with enduring left hip and leg discomfort ultimately diagnosed as avascular necrosis (AVN). She previously underwent intraosseous direct anterior arthroplasty (DAA) of the left hip during the removal of orthopedic artifacts. Despite enduring hypertension, severe trochanter dislocation, and prosthesis fracture, she recovered and required additional surgery to address the dislocation and fracture. This case underscores the challenges in diagnosing and treating AVN, emphasizing the importance of meticulous postoperative care and a multidisciplinary approach. Challenges highlighted by AVN include delayed diagnosis, intricate surgical procedures, and the potential need for further interventions due to hardware complications and infection as seen in this patient.

## Introduction

Direct anterior arthroplasty (DAA) of the hip represents a significant advancement in orthopedic surgery, offering quicker recovery times and reduced postoperative discomfort as a tissue-sparing approach [1]. This method allows for hip joint access through the intermuscular plane without the necessity of a femoral incision, potentially lowering the risk of fracture and facilitating faster healing compared to conventional posterior approaches to the pelvis [2]. However, despite its benefits, DAA poses specific challenges, especially when managing patients with pre-existing orthopedic conditions such as avascular necrosis (AVN).

AVN, also known as osteonecrosis, is a condition characterized by the death of bone tissue due to a lack of blood supply. This lack of blood flow deprives the bone of essential nutrients and oxygen, leading to the breakdown of bone structure and eventual tissue death. AVN presents substantial diagnostic and therapeutic hurdles, particularly in the context of DAA hip replacement [3]. The presence of AVN complicates surgical planning and execution due to alterations in anatomical structures and the heightened risk of complications such as graft failure or delayed healing [4]. Furthermore, patients with prior surgeries, especially those involving bone artifacts, show altered wound tissue integrity. This emphasizes the need for closer monitoring of metabolic factors and increased susceptibility to infections. These considerations may significantly impact the selection of surgical techniques and postoperative management strategies.

The advantages of DAA may encompass enhanced mobility and accelerated postoperative recovery, primarily attributed to the preservation of hip structure and minimal tissue damage [5]. However, these benefits are not devoid of complications, particularly evident in cases of AVN with prior hip surgery. Achieving optimal outcomes for patients mandates a comprehensive grasp of their medical background, impeccable surgical technique, and the ability to anticipate and effectively manage potential complications.

## Case presentation

A 52-year-old female patient was admitted to the emergency department reporting chronic discomfort localized to the left hip and leg. She rated the pain as a 7/10 on the pain scale. The pain was noted to worsen with movement and had shown partial relief with prior interventions, including non-steroidal anti-inflammatory drugs (NSAIDs) and heating pads. The patient's medical history revealed emphysema leading to respiratory failure three months preceding admission, left hip AVN, hypertension, recurrent back and leg spasms, a history of brain aneurysm, spinal cord tumor requiring surgical resection, total hysterectomy, and bilateral mastectomy. It was suspected that her discomfort was due to her history of AVN. The patient had failed conservative management, leading to the decision for surgical intervention.

Prior to surgery, the patient exhibited restricted range of motion in the left hip, characterized by a flexion contracture of approximately 30 degrees and limited internal and external rotation. These limitations were attributed to the presence of significant scar tissue within the joint capsule, stemming from the patient's surgical history.

Upon patient identification, the operative site was delineated, and informed consent was obtained. Subsequently, the patient was transferred to the operating room, where general endotracheal anesthesia was administered. Following sterile preparation and draping of the left hip, a Heuter approach was employed. The tensor fascia was incised along the muscle fibers and retracted laterally, while the rectus muscle was retracted medially. Identification and ligation of the circumflex vessels were performed, and hemostasis was achieved. A circumferential exposure of the capsule was attained, and a capsulotomy was executed.

**Figure 1:**
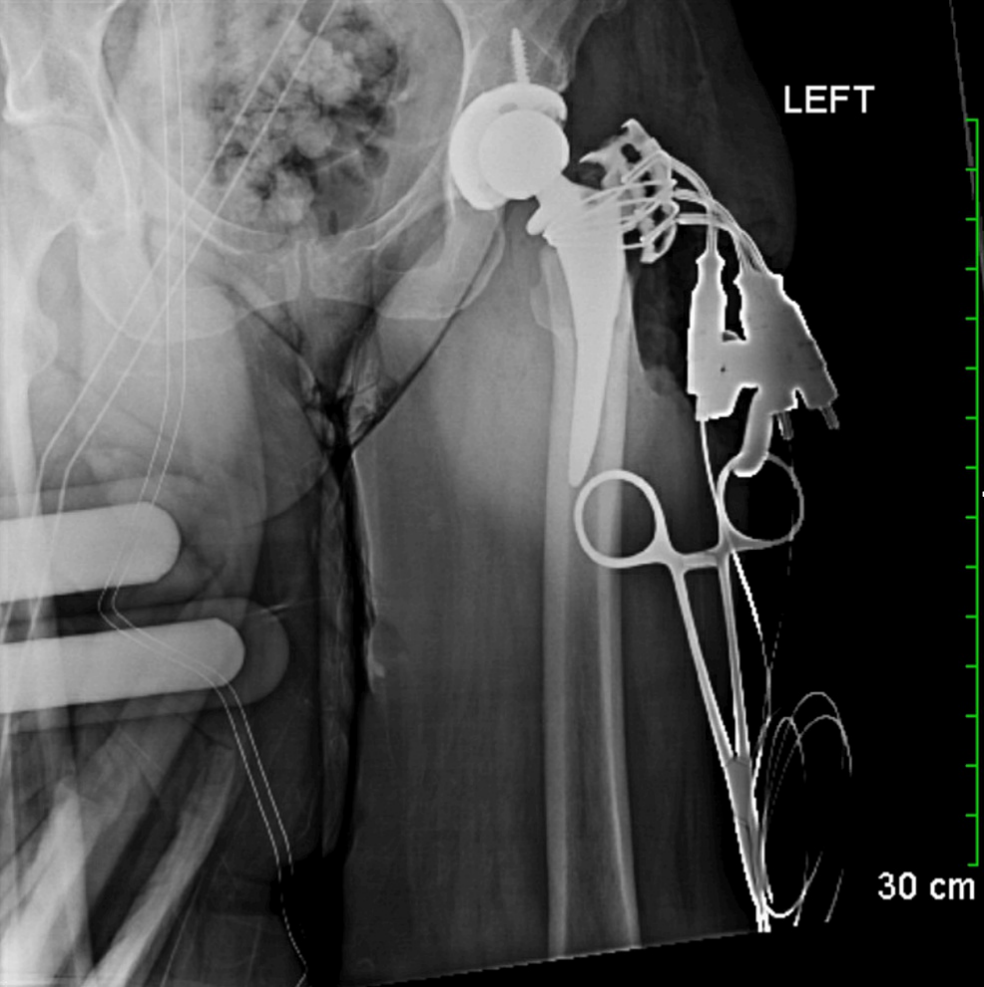
Radiographic confirmation of left hip prosthesis positioning. No evidence of fracture or dislocation observed.

Intraoperatively, after an initial capsulotomy, further releases were required around the medial neck to the acetabulum due to the tightness of the hip. Two femoral neck osteotomies had to be reperformed to facilitate the passage of acetabular reamers. During leg extension, a greater trochanteric fracture occurred necessitating additional intervention. The surgical approach involved a Heuter approach with circumferential exposure of the capsule and femoral neck osteotomy followed by femoral head removal and reaming to appropriate size. A 50mm component was then impacted in place, secured with a screw, and a liner for a 32mm head ball was placed (Figure 1).

Attention was then turned to the femur, which was brought up in the wound with extension, external rotation, and abduction maneuvers. A posterior neck was used as a reference for version, and a rotational-stable stem was placed with subsequent trials to balance leg length and femoral offset. The final stem and head ball were secured, and the hip was reduced. Closure involved irrigation, capsular injection with local anesthetic, and multilayered closure of the capsule, fascia, and skin.

**Figure 2:**
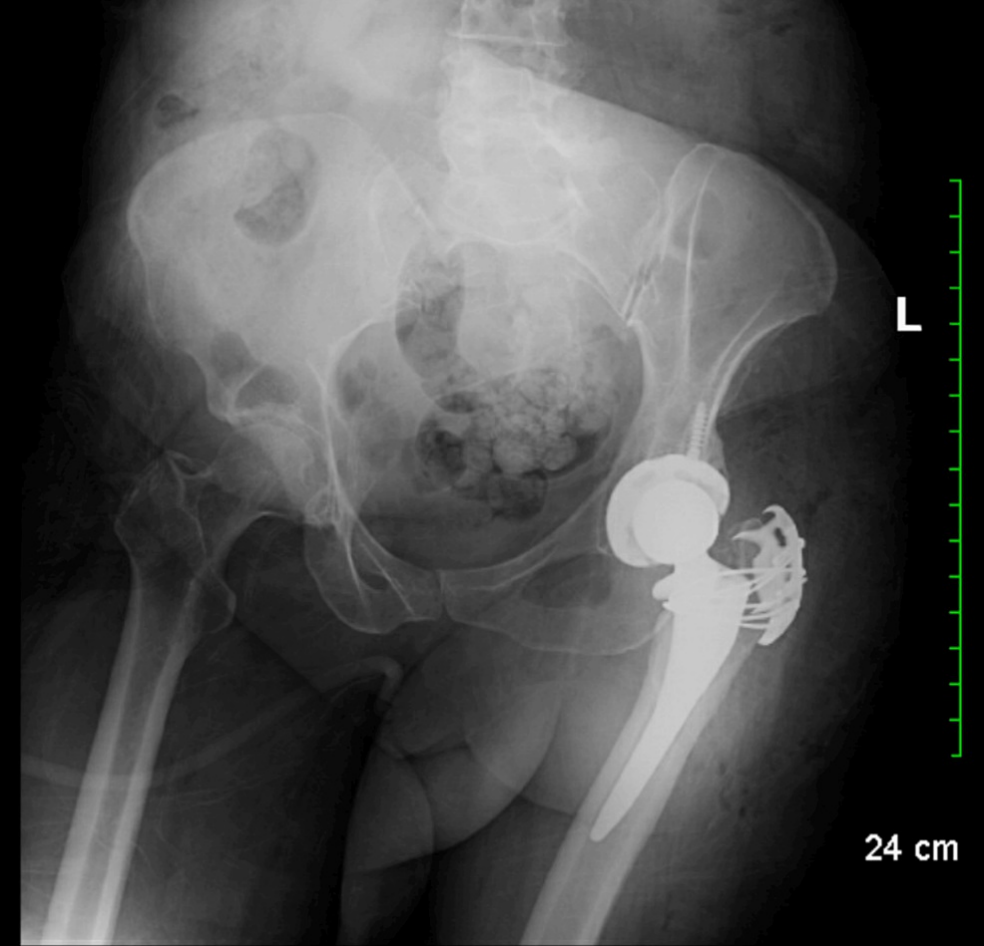
Postoperative radiographic confirmation depicting the finalized left hip prosthesis installation. No indications of fracture or dislocation observed.

After completion of the primary procedure, a greater trochanteric fracture was identified and managed with open reduction internal fixation using a posterior lateral approach. Provisional fixation with a K-wire was followed by application of a trochanteric reattachment device with cables. Closure was performed with irrigation, layered closure, and placement of sterile dressings (Figure 2). Postoperatively, the patient was planned for hip abduction brace and abduction pillow use until the brace arrived, with a weight-bearing plan and restrictions in place.

Following the revision procedure, the patient experienced intraoperative and immediate postoperative hypotension necessitating admission to the intensive care unit (ICU). Management involved the administration of levophed and fluid resuscitation for a short duration to stabilize her blood pressure. Laboratory assessments indicated a hemoglobin level of 5.4 g/dL, which was lower than her pre-operative hemoglobin level of 8.7 g/dL, prompting the administration of two units of packed red blood cells (PRBCs). Pain control was achieved through the utilization of opioid analgesics, including Dilaudid and Demerol.

**Figure 3:**
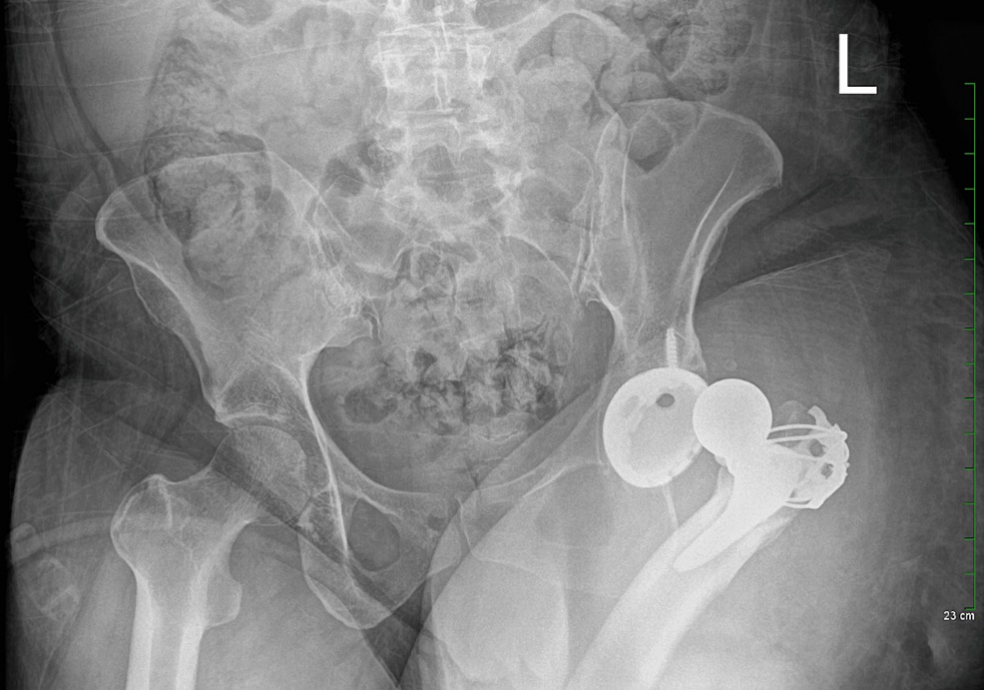
On radiographic examination, the prosthetic left hip demonstrates dislocation characterized by lateral displacement, along with foreshortening of the femoral component.

On the sixth day postoperative, the patient presented with a two-day escalation in hip and thigh pain, alongside increased hip flexion contracture and an inability to abduct her legs. Additionally, she noted the onset of a leg length discrepancy over the past few days, accompanied by severe pain exacerbated by movement. Physical examination revealed a shortened leg length, with the hip flexed and internally rotated. Following evaluation, morning x-rays revealed symmetric hip offset but a slight leg length discrepancy, with the operated hip appearing shorter by a few millimeters. Although the acetabular cup exhibited appropriate abduction, it was noted to lack sufficient anteversion, prompting reconsultation. Subsequent x-rays on the seventh postoperative day confirmed the diagnosis of hip dislocation (Figure 3).

The patient was scheduled for transfer to the emergency department for revision arthroplasty. Evaluation of postoperative and intraoperative images suggests potential impingement resulting from inadequate anteversion of the cup, combined with a slight leg shortening, which predisposes the patient to instability. The surgical plan included revision of the cup, testing hip stability with an increased offset head ball, and having dual mobility and lipped liners available as backup options. Given the recent cup placement, the consideration of constrained liner placement was deferred. Following surgery, strict adherence to posterior hip precautions was implemented, either through continuous bracing or the use of a hip abduction pillow during rest. Additionally, the patient's hip flexion contracture heightened the risk of dislocation and was closely monitored postoperatively.

In the operating room, under general endotracheal anesthesia, the patient received preoperative antibiotics according to protocol and was positioned laterally with appropriate padding of bony prominences. Using the previous incision site, a precise incision was made down to the iliotibial (IT) band, which was then incised along its fibers, followed by division of the medius maximus tendon.

The claw plate previously implanted demonstrated satisfactory fracture alignment, however, loose cables necessitated removal using a cable cutter. Dissection was then continued posteriorly to the femur, facilitating the delivery of the dislocated hip into the surgical field. The femoral head ball was carefully extracted from the trunnion using a bone tamp, revealing a well-preserved trunnion and approximately 20 degrees of anteversion, consistent with the patient's anatomy. Furthermore, the hip stem displayed secure press fit.

Focus was directed towards the acetabulum, involving repositioning of the cup to enhance abduction and version. Following irrigation, a trial with an augmented head ball was conducted, which significantly improved stability, allowing flexion up to 90 degrees and internal rotation without impingement. The hip demonstrated satisfactory stability during extension and external rotation. Subsequently, the acetabular cup was secured with a dome screw, and a neutral liner was inserted. A larger head ball was then impacted onto the femoral trunnion, resulting in successful reduction of the hip with maintained stability.

**Figure 4:**
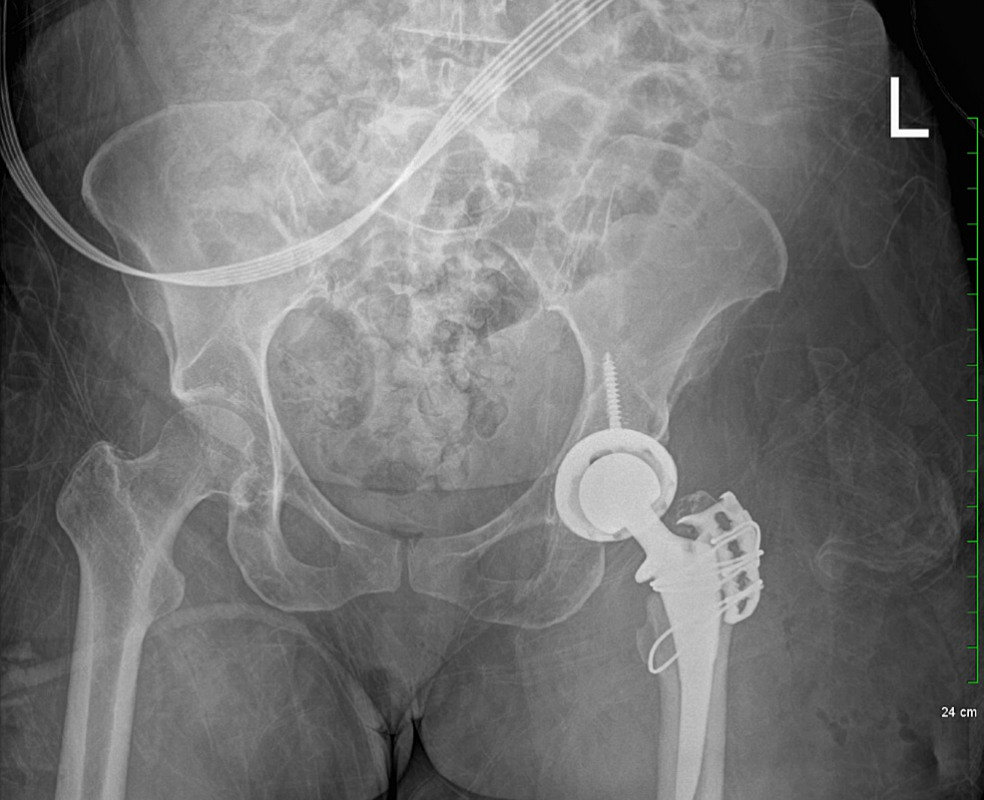
Postoperative radiograph showing near-anatomic alignment of left hip replacement components.

Subsequently, the claw plate was repositioned on the greater trochanteric fracture. Closure of subcutaneous tissue followed using absorbable sutures, and the skin was then closed with a monofilament suture. The patient was then transferred to the recovery room in a stable condition (Figure 4).

In the subsequent days, the patient's condition exhibited improvement, characterized by the stabilization of blood pressure and resolution of hypotension. She appeared comfortable with a dressing applied to her left hip, and no signs of acute distress were observed. Vital signs remained stable, with a blood pressure reading of 131/71 mmHg and oxygen saturation of 97% on room air. The patient reported difficulty sleeping, initially managed with hydroxyzine and subsequently transitioned to Valium 2 mg at bedtime, a medication previously taken at home. Although the patient continued to experience some hip pain, clearance for transfer to the Transitional Care Unit (TCU) for further rehabilitation was provided by orthopedic and rehabilitation services.

## Discussion

The primary management of AVN, particularly in patients with multiple comorbidities and prior surgical history, presents a complex clinical scenario that necessitates a comprehensive understanding of the disease process and a multidisciplinary approach to attain optimal outcomes. This case underscores the significance of tailoring interventions to individual patient needs within the context of surgical management for AVN, along with the subsequent challenges encountered during postoperative rehabilitation.

AVN manifests as bone tissue demise due to inadequate blood supply, resulting in pain and joint dysfunction [6]. The pathogenesis of AVN encompasses multiple factors including trauma, steroid administration, excessive alcohol consumption, and specific medical conditions, all of which can compromise bone blood flow [7]. In this case, the patient's extensive medical and surgical background, including spinal cord injury and prior hip surgeries, likely contributed to AVN development in the left hip. Despite these risk factors, the decision to pursue DAA was motivated by the potential advantages of this surgical technique, such as shortened recovery periods and diminished postoperative discomfort [8]. This raises inquiries about how clinicians can adeptly incorporate patient histories into surgical strategizing, balancing efficiency with the possibilities offered by emerging technologies such as predictive analytics and personalized medicine.

Patients with AVN often encounter prolonged rehabilitation, heightened pain, and a greater likelihood of functional limitations compared to their counterparts without AVN due to the compromised biomechanical and structural integrity of the affected hip joint. The diminished vascularity and compromised bone quality associated with AVN can lead to delayed healing, prolonged recovery periods, and increased discomfort during rehabilitation. Additionally, the risk of implant loosening and perioperative fractures further exacerbates the challenges faced by these patients, necessitating more intensive postoperative management. As a result, individuals with AVN may experience significant difficulties in regaining mobility and achieving optimal functional outcomes compared to those without this condition.

The challenges encountered during the surgical management of this patient underscore the complexity of AVN treatment in individuals with pre-existing conditions [9]. The occurrence of significant trochanteric dislocation and prosthetic fracture during surgery highlights the heightened susceptibility to complications in this patient demographic. These complications include infections, dislocations, implant loosening, thromboembolism, leg length inequality, and nerve injuries [10]. Complications tended to peak within the first three months post-DAA, emphasizing the critical need for vigilant monitoring during this period [11].

Postoperative rehabilitation for patients with AVN, particularly those with multiple surgeries and comorbidities, is crucial in trying to achieve the best functional recovery outcome. Given the intricate nature of these cases, a collaborative interdisciplinary approach to postoperative care, involving orthopedic surgeons, physical therapists, and other specialists, is imperative. Individualized rehabilitation protocols, taking into account the patient's unique needs and medical background, are vital for facilitating recovery and mitigating complications [12].

Patient-specific factors, such as comorbidities and prior surgical history, wield considerable influence over the occurrence and management of AVN. These factors significantly shape the selection of surgical techniques, the risk profile for postoperative complications, and the rehabilitation strategies employed. Given the multitude of considerations, patients are prompted to grapple with the ethical considerations of risk communication and decision-making processes, seeking a delicate balance between potential benefits and risks associated with surgery. This necessitates healthcare professionals to refine the informed consent process, ensuring comprehensive awareness among patients regarding the realistic expectations of outcomes and risks, alongside the potential benefits of surgical intervention. As illustrated in this case, adopting a multidisciplinary approach encompassing preoperative optimization, personalized surgical intervention, and tailored postoperative rehabilitation emerges as pivotal in effectively managing AVN in patients with complex medical histories [13].

## Conclusions

The surgical management of AVN through DAA underscores both its advantages and potential challenges. While DAA holds the potential for faster recovery and reduced postoperative pain owing to its minimally invasive nature, this case highlights the possibility of significant complications, such as trochanteric and acetabular fractures, particularly in patients with metabolic alterations due to underlying conditions. Collaboration among surgeons, physicians, physiotherapists, and nursing staff is essential in addressing complex patient needs, resolving complications, and enhancing rehabilitation outcomes. This article contributes to the expanding literature on AVN management, giving insights into handling patients with significant comorbidities. It emphasizes the significance of meticulous surgical planning, prompt complication management, and coordinated care pathways to optimize outcomes for patients undergoing AVN surgery, particularly those with complex medical histories. Future research aims to refine surgical techniques, enhance postoperative care strategies, and develop comprehensive treatment approaches for AVN to improve patient outcomes, especially among those with substantial medical complexities.
